# Endoscopic fistula closure using a duodenoscope and the reopenable-clip over the line method

**DOI:** 10.1055/a-2584-1997

**Published:** 2025-05-06

**Authors:** Tatsuma Nomura, Takanobu Mitani, Yuto Ikadai, Hiroki Tanaka, Shimpei Matsusaki, Katsumi Mukai

**Affiliations:** 1 Department of Gastroenterology, Suzuka General Hospital, Suzuka, Mie, Japan; 2 Department of Endoscopy Center, Suzuka General Hospital, Suzuka, Mie, Japan

Closing a fistula using clips with a duodenoscope is difficult 1 . However, if a defect cannot be seen with a straight endoscope, it can sometimes be easily observed by changing to a duodenoscope. Therefore, a reliable defect closure method using a duodenoscope is desired. We devised a method called the “reopenable-clip over-the-line method” (ROLM), wherein complete closure can be achieved by placing clips on one side of the defect edge 2 3 . Herein, we describe the first case of defect closure using ROLM with duodenoscopy.

The patient was a man in his 60s who developed a retroperitoneal abscess after acute pancreatitis; the abscess and horizontal part of the duodenum were perforated ( Fig. 1 , Video 1 ). Because the perforation site was difficult to observe with a direct endoscope, we attempted to perform a simple clip closure using a wire-guided reopenable-clip closure with a duodenoscope 4 . However, separation of the wound was confirmed using fluoroscopy 2 weeks after the procedure. The fistula was closed using ROLM with a duodenoscope (JF-260V; Olympus). A clip with a line (SureClip: MicroTech; PE Line: Super X-wire 1.5, DUEL Co.) was placed on the edge of the fistula. The line was then threaded through one of the teeth of the re-openable clip, and the clip was inserted. The line was then pulled while the edge of the fistula was grasped using a clip. After confirming that the clips were brought close together, they were placed. The fistula closed completely after the procedure was repeated. Subsequently, several clips were used to hold the line in place. Finally, the line was cut using the modified locking-clip technique 5 . Complete closure of the fistula was confirmed using fluoroscopic imaging.

**Fig. 1:**
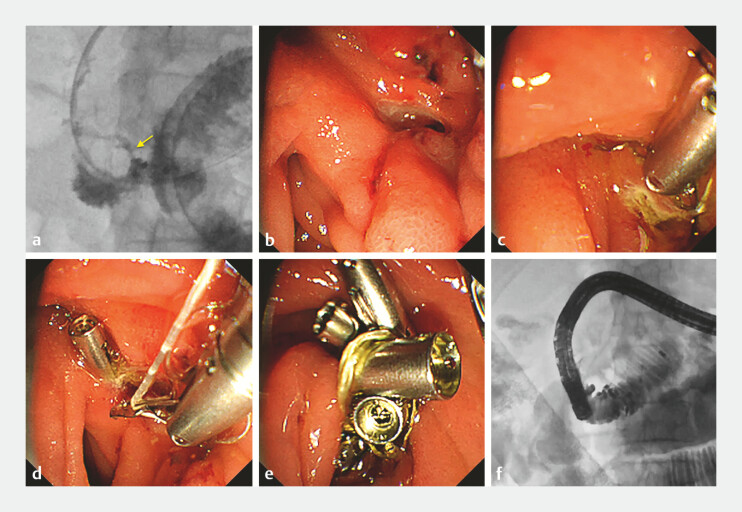
Actual fistula closure using a duodenoscope and the ROLM. **a** Duodenum and retroperitoneal abscess seen on fluoroscopy. Leakage from the duodenal fistula to the retroperitoneum is seen (yellow arrow). **b** Endoscopic image of the fistula using a duodenoscope. **c** First clip with line placed at the edge of the fistula. **d** A re-openable with a line threaded through one tooth is placed on the defect edge. **e** The defect after repeated ROLM. **f** Fluoroscopy after ROLM fistula closure with no leakage into the retroperitoneum. Abbreviation: ROLM, reopenable-clip over-the-line method.

Defect closure using a duodenoscope and the reopenable-clip over-the-line method.Video 1

Endoscopy_UCTN_Code_TTT_1AO_2AI

## Notes

Endoscopy E-Videos https://eref.thieme.de/e-videos
*E-Videos* is an open access online section of the journal *Endoscopy* , reporting on interesting cases and new techniques in gastroenterological endoscopy. All papers include a high-quality video and are published with a Creative Commons CC-BY license. Endoscopy E-Videos qualify for HINARI discounts and waivers and eligibility is automatically checked during the submission process. We grant 100% waivers to articles whose corresponding authors are based in Group A countries and 50% waivers to those who are based in Group B countries as classified by Research4Life (see: https://www.research4life.org/access/eligibility/ ). This section has its own submission website at https://mc.manuscriptcentral.com/e-videos .
